# Why Did I Stop? Barriers and Facilitators to Uptake and Adherence to ART in Option B+ HIV Care in Lilongwe, Malawi

**DOI:** 10.1371/journal.pone.0149527

**Published:** 2016-02-22

**Authors:** Maria H. Kim, Amy Zhou, Alick Mazenga, Saeed Ahmed, Christine Markham, Gerald Zomba, Katie Simon, Peter N. Kazembe, Elaine J. Abrams

**Affiliations:** 1 Baylor College of Medicine Department of Pediatrics, Section of Global Health and Retrovirology, Texas Children’s Hospital, Houston, Texas, United States of America; 2 Baylor College of Medicine—Abbott Fund Children's Clinical Centre of Excellence, Lilongwe, Malawi; 3 Department of Sociology, University of California Los Angeles, Los Angeles, California, United States of America; 4 Health Promotion and Behavioral Sciences, The University of Texas School of Public Health, Houston, Texas, United States of America; 5 HIV Unit, Malawi Ministry of Health, Lilongwe, Malawi; 6 ICAP, Mailman School of Public Health and College of Physicians & Surgeons, Columbia University, New York, New York, United States of America; London School of Hygiene and Tropical Medicine, UNITED KINGDOM

## Abstract

Causes for loss-to-follow-up, including early refusals of and stopping antiretroviral therapy (ART), in Malawi’s Option B+ program are poorly understood. This study examines the main barriers and facilitators to uptake and adherence to ART under Option B+. In depth interviews were conducted with HIV-infected women who were pregnant or postpartum in Lilongwe, Malawi (N = 65). Study participants included women who refused ART initiation (N = 10), initiated ART and then stopped (N = 26), and those who initiated ART and remained on treatment (N = 29). The barriers to ART initiation were varied and included concerns about partner support, feeling healthy, and needing time to think. The main reasons for stopping ART included side effects and lack of partner support. A substantial number of women started ART after initially refusing or stopping ART. There were several facilitators for re-starting ART, including encouragement from community health workers, side effects subsiding, decline in health, change in partner, and fear of future sickness. Amongst those who remained on ART, desire to prevent transmission and improve health were the most influential facilitators. Reasons for refusing and stopping ART were varied. ART-related side effects and feeling healthy were common barriers to ART initiation and adherence. Providing consistent pre-ART counseling, early support for patients experiencing side effects, and targeted efforts to bring women who stop treatment back into care may improve long term health outcomes.

## Introduction

In 2012, the World Health Organization recommended the implementation of Option B+ (B+), which offers all HIV-infected pregnant and breastfeeding women *life-long* antiretroviral therapy (ART) with fixed dose lamivudine-tenofovir-efavirenz [1–4]. Option B+ differed from previous policies by initiating women on life-long ART as soon as they tested positive, regardless of their immunologic or clinical disease stage. Prior to the start of Option B+ in July 2011, only women with low CD4+ counts or with advanced clinical disease were initiated on ART. Studies from Malawi, the first country to implement B+ [5,6], have described impressive increases in ART coverage amongst HIV-infected pregnant women [7–10]. However, long term adherence to ART is needed to optimize prevention and maternal treatment outcomes, and challenges with early loss-to-follow-up (LTFU) [3,11,12], including early refusal and stopping ART, have been described [10,13,14]. HIV-infected pregnant women initiating ART in Malawi were five times more likely to be lost compared to women initiating with low CD4 count [15–17]; in another study, four times more women stopped ART after B+ was introduced [10].

A number of studies have examined potential challenges faced by pregnant and breastfeeding women as they navigate the prevention of mother-to-child transmission (PMTCT) cascade [1,3,18–26]. Barriers commonly noted include low HIV/ART/PMTCT knowledge, stigma, lack of family or partner support, cultural traditions, transport issues, staff shortages, poor staff-client interactions, and service accessibility. Only two published studies have examined these challenges in the context of B+ [1–4], and few studies have examined pregnant women’s reasons for refusing, stopping, or re-starting ART.

The objective of this study was to identify the main barriers and facilitators to uptake and adherence to ART under Option B+, by interviewing women who initiated ART and those who refused or stopped treatment.

## Methods

### Study design, site, participants and procedures

This was a qualitative study of women’s use of ART under B+. Participants were pregnant and postpartum HIV-infected women who attended antenatal clinic (ANC) at one rural and three urban Malawi Ministry of Health (MOH) facilities in Lilongwe District. The combined catchment area of these sites is over 500,000 persons [5,6] with >18,000 persons [7–10] ever starting ART. Over 90% of women attend ANC and receive HIV testing and counseling (HTC). All B+ HIV services, including HTC and ART, are provided free of charge. The sites are supported by the Tingathe program, which, in partnership with the MOH, uses community health workers (CHW) to support retention along the PMTCT cascade [3,11,12]–from identification in ANC through ART initiation, delivery, and final infant diagnosis. CHWs and MOH staff provide HTC and ART education prior to ART initiation. Option B+ was initiated at these sites in July 2011.

**Fig 1:**
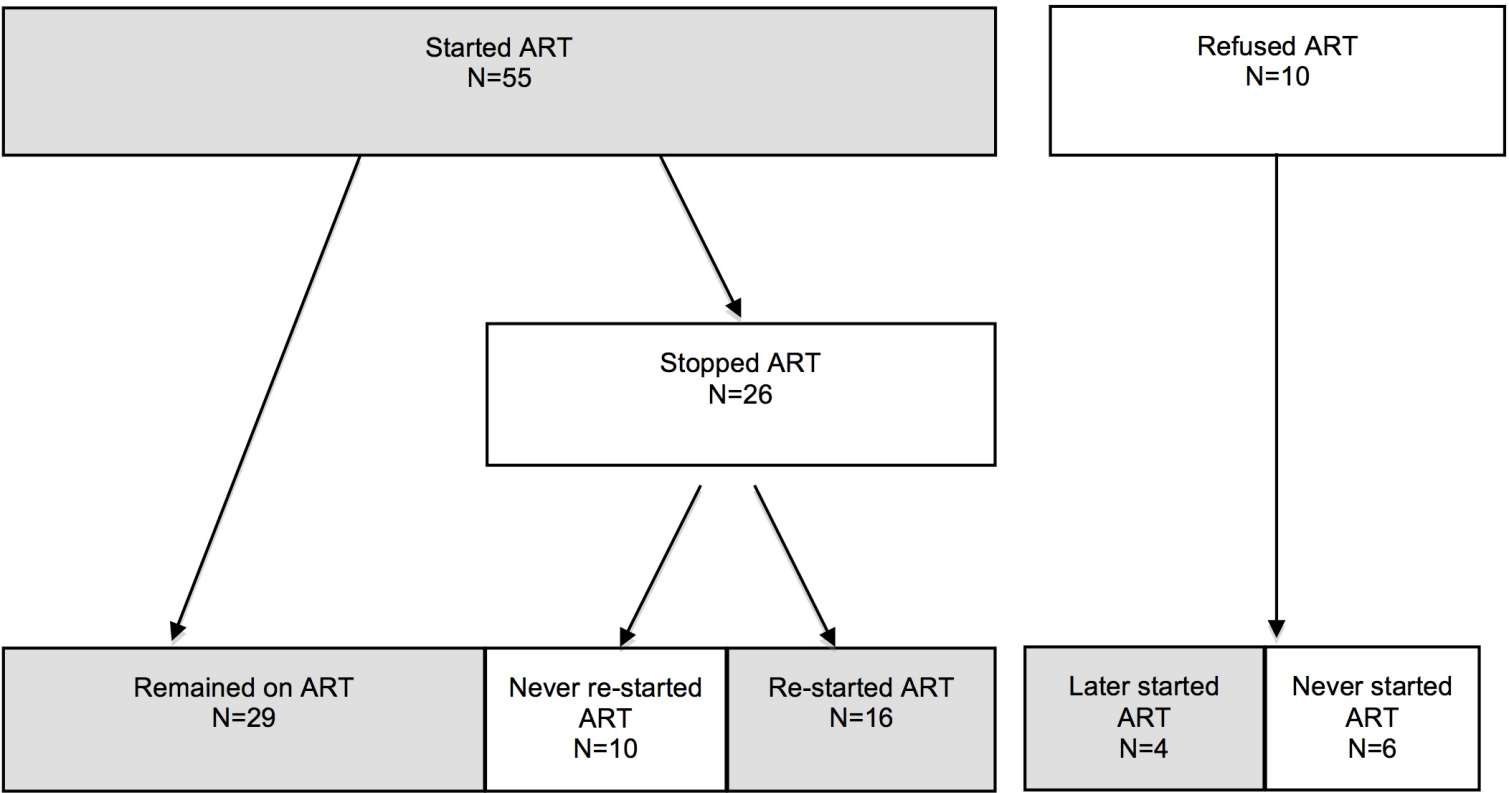
Study sample of HIV-infected pregnant women enrolled in Option B+ services in Malawi.

CHWs identified and extended invitations for interview to potential participants from the Tingathe program. Recruitment took place from July-September 2014. Research assistants explained the study and scheduled interviews. We used purposeful sampling to capture a range of beliefs and experiences thought to be relevant for ART use [10,13,14]. We included women who started ART and then stopped, started and remained on treatment, and those who initially refused ART (Fig 1). CHWs used Tingathe program records to identify women in each category; they contacted potential participants about the project at their home or by phone. Patients’ confidentiality and privacy were maintained. Women were given the option of being interviewed at the clinic, their home, or another location of their choice. The majority of interviews were conducted in the clinic at a private room or secluded area.

Women who refused or stopped ART were oversampled. According to a recent evaluation of the national Option B+ program [16], loss to follow-up 6 months after ART initiation was 24% for pregnant and breastfeeding women. Over half of our sample (36/65) refused or had stopped ART for some time. We oversampled since our focus was to understand why women do not adhere to ART. Stopping ART was defined as not taking ART for any period of time after initially starting ART. Participants were recruited until data saturation when additional interviews yielded little additional new information.

### Ethics

The study was approved by The National Health Sciences Research Committee of Malawi, Baylor College of Medicine Institutional Review Board, and University of California Los Angeles Institutional Review Board. All participants provided written informed consent.

### Data Collection

**Fig 2:**
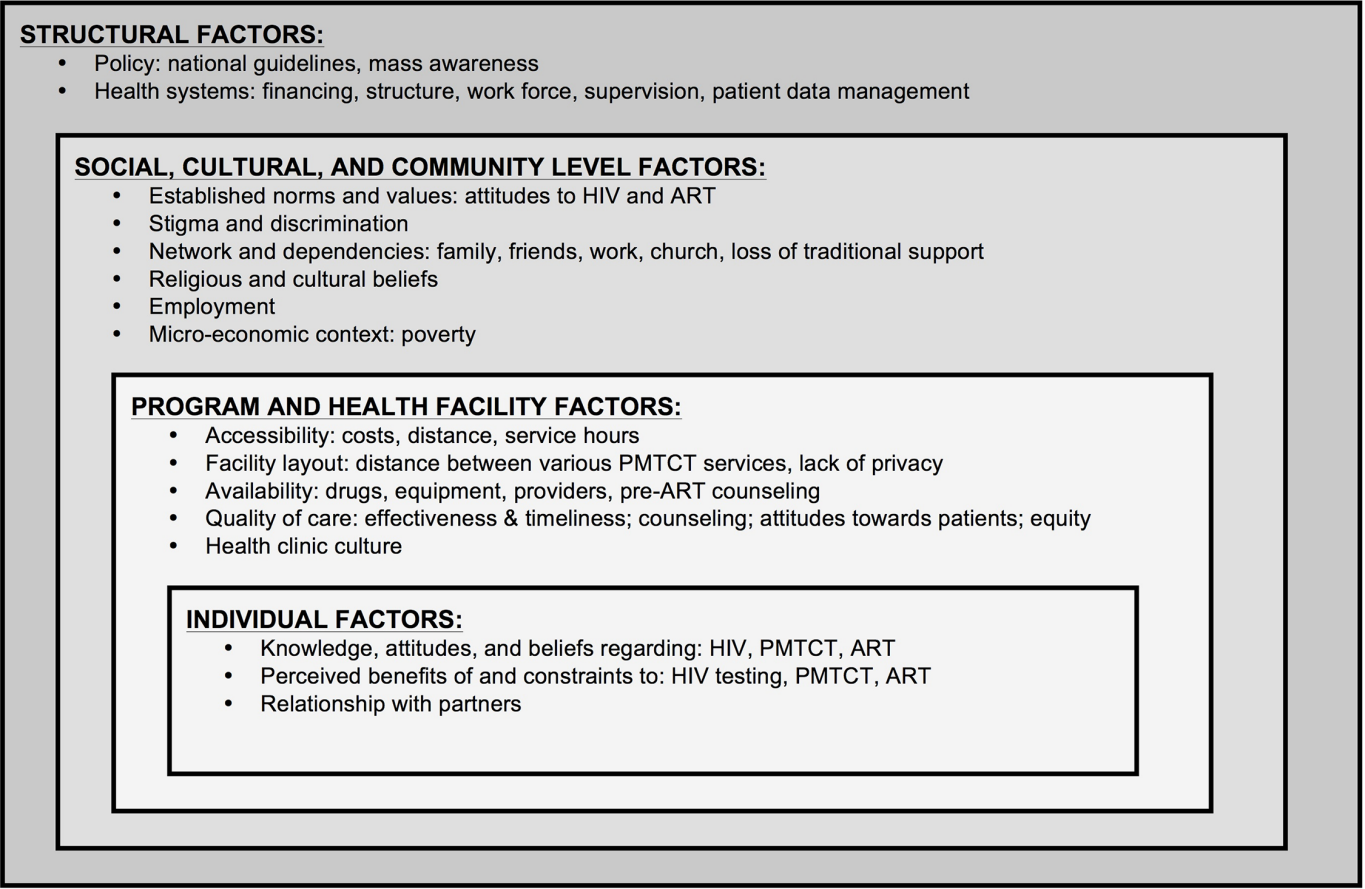
Socio-ecological framework to understand barriers and facilitators of uptake and progression through the B+ PMTCT program cascade. Based on frameworks developed by MacPherson et al, and Mugavero et al.

The interview guide was developed in consultation with health professionals and experts in the field (Malawi, USA) and followed a social-ecological conceptual framework [15–17] (Fig 2) that focused on the barriers/facilitators to care reported in existing literature [10,15,22–28]. The guide was designed to elicit information on potential barriers and facilitators to uptake and adherence to ART under Option B+ and consisted of open-ended questions followed by more specific probing questions. Interview guides were reviewed weekly and modified to incorporate emerging themes. Topics included: (1) experiences with HTC and ANC; (2) starting, refusing or stopping ART; (3) disclosure to partners and others; and (4) impact of religious beliefs and traditional medicines. We noted knowledge of PMTCT and ART by respondents’ reference to counseling messages or to her own desire to take ART to prevent transmission or for her own health. In addition to open-ended questions, we also collected demographic information on participants such as level of education, marital status, water source, lighting, and occupation. The guides were translated into Chichewa and back-translated to English. Questions were pretested with potential participants to ensure understanding and correspondence with the local language and culture. Interviews took place at a location of the participant’s choice. Interviews were conducted in Chichewa by one trained, experienced male, Malawian interviewer, audio-recorded with permission and lasted ~60–90 minutes each.

### Data Preparation and Analysis

Data transcription, translation, review and preliminary analysis started in conjunction with data collection. Interviews were transcribed and translated into English by persons with extensive transcription and translation experience and fluent in Chichewa and English.

Three researchers (MHK, AZ, AC M) performed thematic analysis [1,3,18–26,29]. Transcripts were read multiple times to become familiar with the data, identify patterns, and generate initial codes. Emerging content-driven themes and sub-themes were discussed, and codes refined and categories developed collaboratively. Themes were revised in an iterative and inductive manner. We identified, defined, and re-defined themes as they emerged from the data, and performed coding and re-analysis of previous codes. Concurrent triangulation, or use of multiple sources for verification of findings (interviews with women starting, refusing, and stopping ART), was used to confirm findings [30]. ATLAS-ti was used to facilitate text searching, coding, and analysis. Participant socio-demographic information was analyzed via descriptive statistics.

## Results

From July–September 2014, 65 women completed interviews. Respondents were between the ages of 16–44, with the mean age being 29 years (SD: 7). Most women were living in an urban region (89%) and had completed less than secondary school education (69%). Most women reported a current partnership (78%) and had disclosed their status to their partners (83%) (Table 1). Almost all women had been pregnant before. All respondents who started ART were provided the recommended regimen for B+ of fixed dose lamivudine-tenofovir-efavirenz.

**Table 1:** Characteristics of Study Participants (N = 65).

Characteristic	
**Age**	
Mean, SD, years	29 (7)
Range, years	16–44
**Education**, n (%)	
None	7 (11)
Primary education	38 (58)
Secondary or higher education	18 (28)
Missing	2 (3)
**Marital Status**, n (%)^a^	
Married/relationship	51 (78)
Divorced/single	14 (22)
**Residence**, n (%)	
Urban	58 (89)
Rural	7 (11)
**Water Source,** n (%)	
Pipe/tap	41 (63)
Well/borehole	23 (35)
Missing	1 (2)
**Lighting,** n (%)	
Electricity	18 (28)
Other	46 (71)
Missing	1 (2)
**Occupation,** n (%)	
Housewife	25 (39)
Small business	20 (31)
Piecework	16 (24)
Professional	1 (2)
Missing	3 (4)
**Pregnancy,** n (%)	
First	2 (3)
Subsequent	63 (97)
**Number of Children**	
Mean, SD	3.2 (1.8)
Range	0–8
**HIV diagnosis,** n (%)	
Newly diagnosed during pregnancy	40 (62)
Known HIV status	25 (38)
**Partner Disclosure,** n (%)	
Disclosed	54 (83)
No disclosure	11 (17)
**Disclosure to others,** n (%)	
Disclosed	50 (77)
No disclosure	15 (23)

^a^ Marital status at the time of interview

### Summary of barriers and facilitators to ART

The desire to prevent transmission and improve health was the most influential facilitator for initially starting and remaining on ART. Reasons for initially refusing ART were varied, including: concerns about partner support, feeling healthy, needing time to accept one’s HIV status, and religious beliefs. Side effects were the most commonly reported reason for stopping ART. Lack of partner support was also noted, but less frequently. Despite refusing or stopping ART, many women ultimately decided to take ART for a multitude of reasons including: encouragement from a CHW or others, side effects and other barriers subsiding, decline in health, and change of partner. We found evidence of other barriers–limited knowledge of HIV, stigma, and religious beliefs–but less commonly. Though we acknowledge that several factors might be relevant to women’s use of ART, we have focused on identifying the main barriers to uptake and adherence.

### Reasons for initially starting and remaining on ART

Many of the 29 women who started and remained on ART accepted the rationale behind the Option B+ approach. Participants’ most common reason for initially starting and remaining on ART was the desire to maintain their own health (prevent illness) and prevent transmission to their infants (N = 15).

“My aim is for my life to go ahead, with this disease not having any strength in my body.” (32 years old, divorced, 3 children, urban residence)

“I knew taking them (*ARVs*) each day was something else, but I really wanted to give birth to a normal without a disease child and that was so precious a thing to me.” (32 years old, married, 4 children, urban residence)

In addition, prior health issues facilitated ART initiation and adherence on Option B+. ART was easier to accept because the treatment was seen as a solution to their health issues. A common reason for starting and remaining on ART was feeling sick prior to or at the time of HIV testing. Women believed that their health problems were related to their HIV infection and that ART would improve their health (N = 8).

“Uh it [ART] is good since previously I used to have coughs and cold flu regularly, but all these things stopped; and even feeling general body pains, they no longer happen.” (28 years old, married, 3 children, rural residence)

Others were diagnosed HIV-positive prior to B+ but had not started ART because they had not met eligibility criterion (N = 6). Testing positive earlier had given them time to accept their HIV status, making it easier to start ART now. Three other women who had been previously diagnosed with HIV and refused ART now accepted treatment under B+: two had a decline in health and one gave birth to a child with HIV infection and did not want that to happen again. These life events showed the consequences of not taking ART on one’s health and facilitated the start of ART.

“In my case, I observed for one to begin taking ARVs immediately is good since you protect your child if you are pregnant. For instance, if I started taking drugs immediately, then my child could have not contracted the virus. But just because I didn’t follow the instructions in the beginning, my first child contracted the virus. But when I gained courage to take these drugs, my second born did not contract the virus so I observed ARVs are good and they help a lot in one’s life, yeah.” (30 years old, married, 2 children, urban residence).

### Reasons for refusing ART

Among the 10 women who initially refused ART initiation, reasons for refusing were varied. For many the immediacy of ART initiation under Option B+ was a challenge. Women needed time, whether it was for discussing their status with their partner or personally accepting one’s status. The most common reason for refusing ART was the desire to talk to their partners prior to starting treatment (N = 4). For some, this reflected a fear of disclosure. Because of unstable relationships, they were concerned about their partner’s reaction (N = 2).

“I couldn’t accept them [ART] for the reason of my partner…The child’s father, I can say we could have problems…I was scared that he would be mad and also our marriage could be jeopardized.” (23 years old, married, 1 child, urban residence)

Another reason was difficulty accepting the need for treatment when feeling healthy (N = 2). Women wanted to wait until their health declined before initiating. Others simply wanted more time to process their test results and discuss their condition with their husbands and families (N = 2).

I: Last Monday they told you to start, right [P: Mm] but you refused.

P: Eeh I wanted my relatives to know about my status, I can’t just receive the drugs and take them home while I came here for antenatal services, they would be surprised….

P: No, that cannot happen because I was distressed on that day only that’s why I have come here today [I: Mhm]. Now my heart is relieved since my relatives have counseled me to come and accept to stay in care [I: Mhm] that I should not be worried about that.

I: Okay so you were distressed?

P: I am human, I was healthy then all of a sudden this thing, it’s difficult.

(26 years old, in a relationship, 1 child, urban residence)

One woman described wanting to talk with her husband before initiating ART. Her and her husband tested positive earlier and accepted their status. However, she felt it was important to inform him since they are “one body” and “nobody [should] do things in secret.” She explains:

“So I thought it was a good idea to start taking ARVs, but since my husband didn’t know that I went to the hospital to start on ARV treatment; I just went for antenatal services. So that’s why I told the doctors at the hospital that in my case, it is not a big problem, but since I stay with my husband–who brought me from the village to town and we are one body–there is a need to inform him first.” (28 years old, married, 1 child, urban residence)

We found that religious beliefs did not play a significant role for most women. Only one woman refused because she believed that God, not healthcare providers, would tell her when she needed to start treatment.

Another woman refused, expressing that she was simply not interested in ART at this time; she said, "I just wasn't serious with it…" (24 years old, single, 2 children, urban residence)

### Reasons for stopping ART

Side effects were the most commonly reported barrier to ART adherence. Half of the 26 (N = 13) respondents who stopped ART did so because of side effects. Almost all women who started ART (N = 59) experienced side effects, which included dizziness (78%), nausea or vomiting (17%), nightmares (17%) and hallucinations (9%). Most women experienced multiple side effects at once (55%). Side effects often lasted two weeks (median duration of symptoms was 13 days, IQR 6–32). While most women continued ART despite side effects, some stopped because side effects made them feel worse than before they started treatment. Side effects made some women question the efficacy of ART. One woman, for instance, felt that the medicine made her feel so weak that she was unable to do basic household chores:

“Yea it was weakening me a lot and I could not even do my household chores to the point I concluded that the doctor was lying… later I went to the hospital and told them… the moment I take the medicines I become very weak, look I am very sick. Then later I went to the hospital and told them look I am totally weak. The moment I take the medicines, I become very weak.” (30 years old, married, 4 children, rural residence)

Some of the women who had problems with side effects also expressed challenges with food security (7/13). Women reported worsening of side effects when they took ART without food and they stopped ART occasionally when they did not have enough food (N = 4). Woman may accept ART for life, but have poor adherence during times of food shortage and side effects become too severe to manage.

“While the first drugs, I sometimes didn’t have food to eat but I was able to wake up energetically. But with the new drugs, I usually feel hungry and I feel dizzy when I have not taken any food…Yea I just hit the walls, you feel dizziness or hungry, but when I have eaten something I become very well, yeah. And sometimes you can think I am sick, but I am, the only problem is hunger. Yeah that’s what happens.” (38 years old, married, 4 children, urban residence)

The lack of partner support was another important barrier to ART adherence (N = 6). Women reported fear of disclosing their status to their husbands (N = 2). One woman hid ART from her husband at her mother’s house and stopped treatment when her mother moved. Some women faced obstructive behavior from their husbands, such as throwing away the pills (N = 3).

“When I receive the drugs here, he was throwing the drugs away but I was still coming here to collect those drugs and I started hiding them from him but once he finds them he could take them and throw them away such as that he moved…and I am staying alone.” (21 years old, divorced, 1 child, urban residence)

Although partner support was factored into women’s decision making, in most cases it was not the main consideration. Many women did not return to the clinic even though their partners accepted their status (N = 17). One woman, for instance, took the money her husband gave her for transport to the clinic and spent it on other things. Overall, 44 partners accepted their wives’ status, and often reminded her to take ART every night, whereas 10 partners had overtly negative reactions, such as throwing away pills or divorce (11 did not disclose). Women also restarted ART despite their husbands’ obstructive behavior; two restarted after divorce (N = 2), and one considered divorce if her husband’s behavior did not change. One woman described how her health was more important than keeping the marriage:

“What happened was that when he discovered the drugs you know my husband is violent and he takes alcohol so…I said it’s better I keep on taking drugs because it is my life, I shouldn’t die in the name of marriage. If he gets ill maybe he will receive drugs. In my case, I used to go and receive; and I told my mother, so she encouraged me to continue getting them because I have to raise my children even if our marriage ends.” (29 years old, married, 3 children, urban residence)

A couple of women stopped ART because they had forgotten or lost their pills (N = 2). One woman was traveling and forgot her pills at home. Another lost her ART when her mother fell sick, and she did not start again because there were no negative consequences to her health after stopping.

### Other barriers to ART retention

We examined evidence of other barriers–stigma, religious beliefs, transport, clinic related and knowledge of PMTCT/ART,–but found they were less salient for women’s adherence.

Most respondents acknowledged that stigma exists in their community, mostly as gossip (N = 42). Respondents were hesitant to widely disclose their status to community members for fear of being talked about.

“There are some people who do not speak well about others…they scorn by saying look at her! ‘She broke’…they say ‘she has broken.’ So I don’t like that kind of saying. When I look at the people I feel sorry for them but to them. They think it is something, a good talk with their friends, forgetting that one day they may be found HIV positive as well.” (31 years old, married, 3 children, urban residence)

While stigma is acknowledged, there were few reports of overt discrimination and only one respondent refused or stopped ART because of stigma (N = 1); she reported stopping because her sister ridiculed her about her pregnancy and HIV status.

Other infrequently reported barriers included religion, transport and clinic related challenges. One woman stopped ART for one year to try healing through prayers. She explained that after reading a biblical verse about God’s ability to heal, she “decided that God would be the one to heal [her] (30 years old, married, 4 children, urban residence).” Another woman stopped because she moved and did not have money for transport to the clinic and another because she was turned away for not coming on an ART clinic day. One woman reported feeling “lazy” to return to the clinic to refill, especially given that she felt fine without ART despite being off treatment for one year.

Most respondents had knowledge of PMTCT and ART (N = 55), while others did not recall receiving this information (N = 10). Respondents forgot the counseling message, or mentioned nevirapine but not ART as the drug that prevents transmission. We found few instances of overt skepticism toward counseling messages (N = 3). Of the women who had insufficient knowledge (N = 10), three had refused and five were lost to care.

### Reasons for re-starting ART after refusing or stopping

Many women (N = 22) started ART after initially refusing or stopping– 4/10 who refused started, and 16/26 who stopped re-started by the time of the interview. Women started or re-started ART about 2.5 months after refusing and about 1 month after stopping. Women reported different periods of side effects, with most reporting that symptoms went away in a couple of months (median 13 days, IQR 6–32). An important facilitator to starting or restarting was encouragement from CHWs (5/22). All women were part of the Tingathe program and had the same access to CHWs. CHWs provide monthly home visits to check on women’s use of ART and provide treatment support. Almost all women expressed positive experiences with their CHW. CHWs explained the side effects, reminded women to think of her child, counseled husbands, and encouraged them to re-start. CHWs also continued to visit women who had unsupportive partners–usually when partners were not home. Encouragement from CHWs as well as others helped women re-start ART. For instance, one woman noted that encouragement from her CHW and husband helped make sense of their HIV status and start ART.

“It was something I didn’t believe that it could happen to me…. so they (CHWs) were the ones who came to encourage me that I should accept the results…So after sometime, my husband also advised me to accept the results. ‘Whatever the doctors instruct you, you need to follow them….’ That’s when I started taking the drugs.” (27 years old, married, 1 child, urban residence)

Experiencing a decline in health was another reason for starting or restarting ART (N = 3). One woman, who had quit three times before due to side effects, started again after feeling sick (P015).

I: So what happened that you should start drinking [taking ART] again?…

P: I was sick…I was feeling cold even though it is sunny. I was feeling some piercings in my limbs. I felt like someone has a needle and he is piercing me…coming here, they told me that I should also learn. They said that because I started medication and stopping. I should learn. And after that then I started drinking the medicine. (24 years old, married, 2 children, urban residence)

Another reason for starting or restarting ART was fear of future sickness (N = 2). While women felt fine at the time, they were worried that they would become sick if they continued without ART. One woman who had stopped ART for month explains:

“I was afraid I would get sick…feeling like body pains or fever, yeah, so I thought it is better to start taking drugs.” (44 years old, widow, 4 children, urban residence)

For women who stopped ART, a key facilitator to restarting was the subsiding of side effects (N = 5); most of these women had been non-adherent during food shortages citing that side effects were worse when ART was taken without food (N = 4).

P: I just decided on my own: “I will stop taking the medicine; after I give birth, I can start again.” Because it was like, once I take it, I could vomit all the time. So I just said, “I will stop” …

P: And then started again in July.

I: So when you started again, did you get sick again?

P: No, even if I were to take the whole bottle a day.

(24 years old, married, 5 children, urban residence)

Changing partners also facilitated re-starting ART (N = 2). Women were able to take ART after the divorce of a previously unsupportive partner. One woman who had stopped ART because of transport issues after moving, restarted one month later after she found a clinic that was closer to her new home. One woman, who stopped because of religious beliefs, also restarted in part due to her religious beliefs because she wanted to combine prayers with ART to protect her unborn child. Another woman decided to restart after gaining encouragement through her prayers.

I: So when you were told your test results that you are tested HIV positive, what came into your mind that time you were disclosed your results?

P: Uh nothing, it was like I didn’t believe the results–that it can happen to me. …In the first days, I was really anxious. But because sometimes I go to church to pray, then I discovered that encouragement began to grow because I was going to church to pray. So now I accepted it and I am stable, I don’t think about it often but early days it was difficult for me to stabilize, yeah (27 years old, married, 1 child, urban residence)

## Discussion

This is one of the first studies to examine barriers and facilitators for ART initiation and adherence amongst women initiating lifelong ART in the context of Option B+. Furthermore, to our knowledge, this is the first study that examines the experiences of women who stopped, or did not initiate treatment in order to understand barriers and facilitators to ART use. As such, this study offers important and unique insights into the reasons for initiation and non-adherence in the context of B+. We found that in the context of B+, inadequate time to prepare to initiate ART as well as side effects emerged as more significant barriers as compared to previous studies on barriers and facilitators in non-B+ contexts. Other previously described barriers such as partner support remained but we found a more nuanced power dynamic. We also learned that many women eventually started or restarted ART after initially refusing or stopping. The decision to refuse, stop, restart, or remain on ART was influenced by multiple factors. However, the main reasons women reported for initially refusing ART were needing more time to think, concerns about partner support, and feeling healthy. The main reasons for stopping ART were side effects and lack of partner support. For those who restarted ART after initially refusing or stopping community health worker support, side effects subsiding and a decline in health facilitated this. The main facilitators of remaining on ART were the desire to prevent transmission and future illness, as well as treat past or current illness.

Of particular concern are early ART-related side effects that appear to influence women’s ART adherence. Half of the women who stopped ART cited side effects as their main reason. Women expressed feeling less healthy after taking ART. This may in part elucidate findings from Malawi that reported that women who started ART in the context of B+ were 5 times more likely to be lost to follow-up as compared to women who started for their own health [16]. Side effects may have been identified as a more important barrier in our study because unlike the prior studies [1,3], we interviewed women who had stopped ART. Importantly, although almost all women experienced side effects, only some chose to stop because of them. Increasing evidence suggests that EFV-related toxicities are related to high plasma levels which are influenced by a variety of factors including pharmacogenetics and pharmacokinetic characteristics [31–34]. It is possible that women who stopped treatment were slow metabolizers or of lower body weight and experienced more severe side effects secondary to higher plasma levels. Economic concerns such as impact of side effects on the ability to earn a living or food insecurity were also influencing factors. While our study design did not include systematic sampling according to levels of food security, it emerged in our analysis that the availability of food impacts women’s capacity to stay on ART. This finding is especially relevant for low-income countries, like Malawi, that struggle with food security. Additional research would help clarify these findings. Meanwhile, given that the most commonly experienced problems were those that are short-lived, pre-ART counseling about temporary efavirenz related side effects and focused support during this early period might help women process the experience and encourage them to adhere to ART. Our findings also suggest a growing need to identify alternative drugs with a lower burden of toxicities that are safe, efficacious and feasible to use during pregnancy and the postpartum period in high HIV burden settings.

With Option B+ more women who *feel* healthy are initiating lifelong ART. We saw the emergence of the pivotal influence of a subjective feeling of health, which has previously not been noted as a major influence affecting adherence amongst HIV-infected pregnant women [18,19]. A common reason for starting ART was feeling sick, a common reason for refusing ART was feeling healthy, and a common reason for stopping was that ART-related side effects made them feel less healthy. Furthermore, women who remained on ART cited the benefit of ART in maintaining their current state of health. These are important findings as countries move to universal treatment for all individuals with HIV infection as it will be critical to consider counseling messages and support for those initiating at earlier stages of disease.

In our study we found that partner support was repeatedly found to influence women’s PMTCT related decision making [18,19,28]. We also found it to be an important consideration in a woman’s decision to take or not take ART. During the initiation phase, one of the main reasons women refused was because they wanted to consult their husbands and return to the clinic together. After initiation, a lack of partner support could become a clear barrier for adherence to ART. Husbands were reported to refuse to allow their wives to visit the clinic, and to throw away their pills. While we indeed found that partners sometimes prevented women from accessing ART, the relationship dynamic was nuanced with some women stopping treatment despite partner support and others divorcing their partners to be able to take ART. Women had many reasons—such as side effects, needing more time, and feeling healthy—for not taking ART, which were often not related to her partner’s opinion. Women also responded to their partners’ reaction, sometimes ignoring their instructions or divorcing their partners. This is not to propose that unequal power dynamics is not a concerning challenge, but our findings suggest that the power dynamic is nuanced rather than uni-directional and that women are active in making treatment decisions [35]. Following the growing body of research on gender-based violence and gender inequality as determinants of women’s risk for HIV infection [36], more research on how couples understand decisions for taking ART would help clarify the effects of gender power dynamics on ART adherence.

While we find some evidence of other previously reported barriers–in particular knowledge of PMTCT/ART and stigma–they were reported less often. The high levels of knowledge of PMTCT/ART in our cohort may reflect bias in our sampling. Respondents were drawn from a health facility that employs dedicated CHWs to provide support to pregnant and postpartum women. However, while inadequate knowledge was not found to be one of the main reasons for refusing or stopping ART, it may have been a contributing factor. Other studies have also noted that stigma significantly influences women’s choices around ART [18,21,37]. In our study, while women noted that stigma still exists in the community, very rarely did they report that it factored into their decision to take ART. Our findings also suggest that stigma may not be as severe as it was in the past. There were few overt instances of discrimination.

Economic barriers are a commonly noted barrier to care and may be more significant than identified in this study [18,19,21,26]. Only one woman reported stopping ART because of lack of funds for transport. However, our finding that food affects the severity of side effects suggests that economic barriers may manifest as an indirect mechanism that affects ART use. While we gathered demographic information on women’s source of income, water, and lighting, we did not include probing questions to describe in detail her living condition and only reported economic barriers that the women specifically reported on her own.

A key strength of this study was the use of in-depth interviews with not only women who stayed on ART but also those who refused, stopped, and re-started. To our knowledge this is the first study to describe the experiences of women who refused or stopped ART in the context of B+. In addition, we explored several possible barriers and parsed out which barriers appeared to be most salient for ART initiation and adherence in B+ care.

While there is some overlap between our findings and that of research on ART adherence in the general adult population, we argue that particular barriers become more salient for women initiating ART in the context of B+. Prior to B+, women were only initiated on ART once they were immunologically compromised or clinically unwell. Immunologic assessment often took greater than one month. This delay may have provided women with more opportunity to come to terms with their HIV diagnosis and the recommendation to start lifelong ART. With Option B+, immunologic assessment was no longer necessary to determine ART eligibility, since all women became automatically eligible upon HIV diagnosis. This may be why we found that in the context of B+, women expressed the need for additional time to prepare. They wanted time to discuss their status with family members, partners, as well as personal time to cope with their status. Also notable were the number of women who stopped and then re-started ART within the first few months of initiation. These women may not have been ready to start in the first place. Although ART ideally should be started as soon as possible to rapidly reduce viral load and prevent HIV transmission during pregnancy, this need must be balanced with providing women with sufficient time to feel ready to start and adhere to treatment. Targeted efforts to provide support to women during the early period post diagnosis of HIV may help to ensure a smoother transition from ART start to long-term adherence.

Although prior studies demonstrate that side effects may be a barrier to ART adherence amongst the general adult population, our study suggests this may be a more significant barrier in the context of B+. Unlike adults in the general population, under B+ women started ART while pregnant and therefore possibly confounding ART side effects with unpleasant pregnancy symptoms. In addition, many also started ART before they felt clinically unwell. This may have made the side effects more difficult to tolerate.

There are several limitations to this study. Our study population comes primarily from an urban setting, and focused on women initiating ART during pregnancy (not during breastfeeding), and therefore may not be representative of all HIV-infected women engaged in Option B+ care in Malawi. In addition, respondents were at sites receiving retention support from the Tingathe program [12], and therefore previously reported barriers such as knowledge of PMTCT/ART and lack of provider support may have been less influential. Additional qualitative research from other sites would help triangulate and verify the results. Because our sampling focused on categories of ART use, we did not have enough variation in demographic factors such as age, education, and pregnancy (first or subsequent) to analyze how those factors may impact women’s use of ART. Finally, this paper describes what women express to be the most salient barriers. While this highlights the most important reasons for starting and stopping ART, there are likely very rich interaction effects that warrant further exploration.

## Conclusion

With the initiation of Option B+, Malawi has made impressive progress in improving access to PMTCT care. However, this study demonstrates that challenges with uptake and adherence to ART remain and that there are multiple causes. Adequate time and support for women to make the decision to begin treatment, consistent pre-ART counseling, early support for patients experiencing side effects, and timely efforts to bring women who stop treatment back into care may improve long term treatment outcomes.
